# Evaluation of electronic patient-reported outcome assessment with cancer patients in the hospital and at home

**DOI:** 10.1186/s12911-015-0230-y

**Published:** 2015-12-23

**Authors:** L. M. Wintner, J. M. Giesinger, A. Zabernigg, G. Rumpold, M. Sztankay, A. S. Oberguggenberger, E. M. Gamper, B. Holzner

**Affiliations:** Department of Psychiatry, Psychotherapy and Psychosomatics, Medical University of Innsbruck, Anichstr.35, Innsbruck, 6020 Austria; Department of Internal Medicine Kufstein County Hospital, Endach 27, Kufstein, 6330 Austria; Department of Medical Psychology, Medical University of Innsbruck, Schöpfstraße 23a, Innsbruck, 6020 Austria; Leopold-Franzens-University of Innsbruck, Innrain 52, Innsbruck, 6020 Austria

## Abstract

**Background:**

Patient-reported outcomes (PRO) provide a more comprehensive picture of patients’ quality of life than do mere physicians’ ratings. Electronic data collection of PRO offers several advantages and allows assessments at patients’ homes as well. This study reports on patients’ personal internet use, their attitudes towards electronic and web-based PRO assessment (clinic-ePRO and home-ePRO) and the feasibility of these two assessment modes.

**Methods:**

At the Medical University of Innsbruck and Kufstein County Hospital, cancer patients who participated in clinic-ePRO/home-ePRO were asked to complete a comprehensive evaluation form on their personal internet usage, attitudes towards and the feasibility of routine clinic-ePRO/home-ePRO with the Computer-based Health Evaluation System (CHES) software.

**Results:**

In total, 113 patients completed the evaluation form for clinic-ePRO (Ø 45 years, SD 14) and 45 patients for home-ePRO (Ø 58 years, SD 10; 33.1 per cent inclusion rate for this sample). Most patients expressed willingness to complete routine clinic-ePRO assessments in the future (94.7 per cent of clinic-ePRO patients and 84.4 per cent of home-ePRO patients) and to discuss their data with attending physicians (82.2 per cent, home-ePRO patients only). Overall, patients preferred the software over paper-pencil questionnaires (67.2 per cent of clinic-ePRO patients and 60 per cent of home-ePRO patients) and experienced it as easy to use. Only a few minor suggestions for improvement were made (e.g. adjustable font sizes).

**Conclusions:**

The use of clinic-ePRO/home-ePRO was in general shown to be feasible and well accepted. However, to be more inclusive in the implementation of clinic-ePRO/home-ePRO, educational programs concerning their particular benefit in oncology practice potentially could enhance patients’ attitudes towards, and consequently their acceptance of and compliance with electronic PRO assessments.

## Background

Patient-reported outcomes (PRO) regarding symptoms and quality of life (QOL) have become an important part of cancer clinical trials and routine care, as there is strong evidence that clinicians’ ratings alone do not present a complete picture of cancer patients’ symptom burdens [1–5]. Studies emphasise the additional benefit of the use of PROs (improved symptom management [6], better identification of intimate or psychosocial problems [7], enhanced communication between health care professionals and patients [7–9]) and the administration of validated questionnaires via specialized software helps to overcome common implementation barriers to the routine use of PROs (e.g., administrative obstacles, concerns that electronic assessments might use more resources than they save) [10, 11]. Electronic PRO (ePRO) assessment is generally well accepted by patients [6, 12] can be conducted in an efficient and timely manner, and both in clinical settings and in patients’ homes. Web-based home monitoring offers insight into the “black box” of a patient’s condition between hospital visits, a parameter that is systematically overlooked by QOL assessment restricted to the hospital setting. The large number of by now available specialized software solutions for ePRO assessment, predominantly developed within English speaking countries and offering a variety of data collection and educational features [13, 14], mirrors the growing interest in ePRO assessments. However, ePRO assessments require a certain level of patients’ computer literacy; therefore, patients’ habits and attitudes regarding the use of new technologies (devices and technologies, which can be summed up under the umbrella term “new media”, e.g., computers, smart phones, and the internet) are particularly important. Devices for internet access and internet infrastructure are becoming increasingly affordable and easy to manipulate. Nevertheless, some groups (e.g., older, computer-illiterate, or socioeconomically disadvantaged patients) might be put at a disadvantage when data collection is conducted mainly via new media (i.e., computer-based assessments via internet-ready devices), as familiarity with such devices seems to play an important role for implementation of ePRO and patient recruitment [15, 16]. Though, available statistics suggest that actual existing problems with lacking familiarity with or open-mindedness to computer and internet-ready devices might vanish in the course of time. For instance in the Austrian general population in 2014, about 40 % of persons older than 65 years access the internet on a regular basis (a slightly more than tenfold increase since 2002), roughly a third of these using mobile devices [17]. Nearly as many cancer patients aged 66 to 99 years reported the availability of their own internet connection and at least occasional use of the internet [12]. Given the actual technical development, this number can be expected to further increase. Nevertheless, the feasibility and user-friendliness of used software solutions for ePRO assessment need to be evaluated for optimization of applied systems.

### Objectives

This study aims to gather information on cancer patients’ personal internet use and their attitudes towards electronic QOL assessments. Furthermore, we evaluate the user friendliness and feasibility of an ePRO software used in the hospital setting (clinic-ePRO) and its advanced interface [18] for internet-based assessments at home (home-ePRO). Accordingly, the formal objectives of this cross-sectional study are:

- to assess patients’ basic habits regarding the personal use of the internet
- to assess patients’ attitudes towards clinic-ePRO or home-ePRO, and
- to evaluate the feasibility of the clinic-ePRO and home-ePRO procedure.

## Methods

### Sample and procedure

**Fig. 1:**
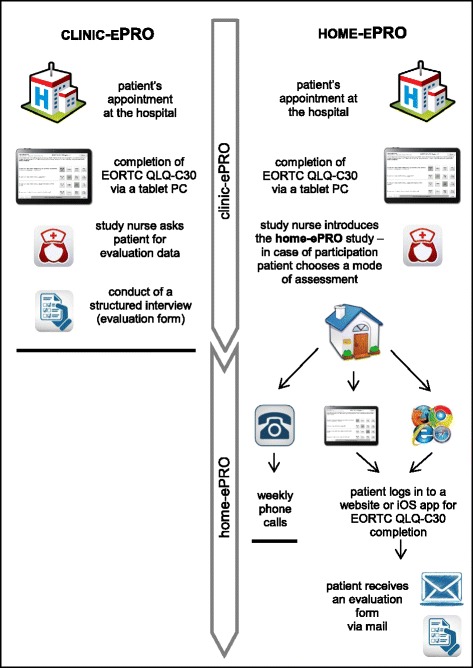
Flow chart describing procedure for clinic-ePRO and home-ePRO

Cancer patients at various departments of Medical University of Innsbruck (urology, neurology, nuclear medicine, internal medicine) and at the Department of Internal Medicine at Kufstein County Hospital were approached before their consultation or treatment application by a study nurse, who explained participation in the electronic QOL assessment (at the hospital or at home; see Fig. 1 for a flow chart of the procedure), and obtained patients’ written informed consent. The requirements for patients’ participation included minimum age of 18 years, diagnosis of oncologic disease, no severe cognitive impairments, and German-speaking. The study was approved by the ethics committee of Medical University of Innsbruck.

### Clinic-ePRO

Patients at Medical University of Innsbruck were asked to complete the European Organisation for Research and Treatment of Cancer Quality of Life Questionnaire (EORTC QLQ-30) while waiting for their appointment with their attending physician. Assessments were completed by the patients themselves using a tablet PC for data entry with the Computer-based Health Evaluation System (CHES). A study nurse provided an initial training if necessary and further assistance if patients reported any problem with the device or the questionnaire. Clinicians had access to patients’ self-reports via their work desktop, but there was no record of whether they actually accessed such data and no advice was given on how to use PRO data for patient appointments.

### Home-ePRO

At the Department of Internal Medicine at Kufstein County Hospital, outpatients receiving chemotherapy were asked to complete the EORTC QLQ-C30 before each treatment application (clinic-ePRO). A tablet PC for autonomous data entry was used, and patients could refer any question to a study nurse. Some clinicians used CHES on their work desktops and/or printed QOL-reports in patient appointments, but no structured guideline was provided for the use of these data.

Those patients already involved in clinic-ePRO completion at outpatient visits were asked whether they wanted to provide information about their QOL at home as well by completing the questionnaire via either a website, with an iOS application (app) (developed by World Direct within an OncoTyrol project, used with Apple iPad2, Apple Inc., Cupertino, California) or a telephone interview. Those patients who chose home-ePRO were given a printout that showed their login data (an individual user name and a secure password), and for web access, the URL of the webpage. If necessary, an Apple iPad 2 (including a preinstalled iOS app) was provided by the hospital as a free loan for the duration of study participation.

### Evaluation

After participating in clinic-ePRO or home-ePRO, patients were asked to complete an elaborated evaluation form. Patients at Medical University of Innsbruck completed the evaluation via a structured interview with a study nurse right after QOL assessment and during the waiting time for their appointment. Participating patients at Kufstein County Hospital received the evaluation form and return envelope via mail. If there was no response within 3 weeks after the first mail, patients received a written reminder with another evaluation form.

The comprehensive evaluation form was especially developed for this study by two members of our working group, incorporating experiences from previous studies [19, 20] as well as topics of scientific and clinical interest. A preliminary version of this form was reviewed by a clinician who gave advice on appropriateness and acceptable burden for patients. Several domains such as personal internet usage at home, private internet activities, device handling, and attitude towards clinic-ePRO/home-ePRO, were addressed. Because evaluation forms were tailored to the mode of assessment (at the hospital or at home), each questionnaire included, in addition to identical items covering core issues, a section of items dealing with specific aspects of clinic-ePRO or home-ePRO. Patients were asked about the mode of data collection, e.g., their willingness to complete QOL questionnaires in the future (at the hospital as well as at home), their preferred mode of administration (paper-pencil, clinic-ePRO, or no preference), and whether they preferred phone calls over home-ePRO. They also rated the perceived usefulness of clinic-ePRO/home-ePRO for informing their physician about their QOL, and were asked about their interest in seeing and storing their own results. Two questions dealt with subjectively perceived advantages and disadvantages of clinic-ePRO/home-ePRO. All questions had predefined answer categories (e.g. similar to the EORTC QLQ-C30 “not at all”. “a little”, “quite a bit” and “very much”, or other categories according to the content of the question) and an additional open answer, if patients wanted to add information/make suggestions.

### Electronic data capture

The Computer-based Health Evaluation System (CHES) is a specialized software for electronic PRO data collection, result calculation, and presentation for use in clinical practice as well as for merging clinical and PRO data for research purposes [18]. We used CHES for both clinic-ePRO in the hospital setting and home-ePRO in patients’ homes; the assessment procedure is outlined above. A study nurse gathered sociodemographic and clinical data from the hospital records and entered them in CHES to link with associated QOL data stored in the CHES database.

**Fig. 2:**
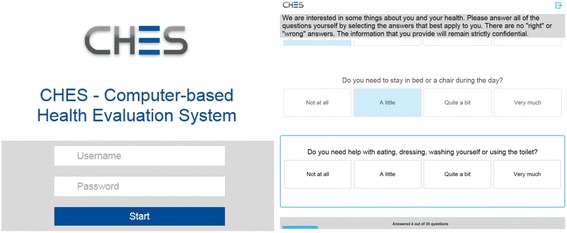
Screens for login to the CHES home-ePRO (left) and for questionnaire completion (right)

The CHES software works with a database stored at each particular hospital server, which patients and physicians access by different means. Physicians can use CHES via their work desktop (for display of patient data as longitudinal or cross-sectional reports, preparation of individual questionnaire sets, and/or making data available for patients online). Patients are equipped with login data for access to questionnaires via a website or an iOS app (see Figs. 1 and 2). Consequently, both physicians and patients use the same software but are accessing different features. For a more detailed description of the features of CHES, please refer to Holzner et al. [18].

### Statistical analysis

Patient characteristics were computed as means, standard deviations, percentages, and ranges. For comparison of mean age of patient groups one-way ANOVA (three groups) and for comparison of distribution of sex *χ*2—Test were calculated (5 % level of significance).

## Results

### Patient characteristics

#### Clinic-ePRO patients

At Medical University of Innsbruck, between February and September 2012, 113 patients (mean age 45.1 years, SD 14.4, range 22 to 81), most of whom (88.5 %) were attending an outpatient follow-up appointment, completed the evaluation form directly after clinic-ePRO assessment (see Table 1). Most common diagnoses were testicular cancer (67 %), glioma (15.7 %) and neuroendocrine tumours (12.8 %). Data on the number of patients approached for evaluation completion are not available.

**Table 1:** Patient characteristics

		Clinic-ePRO at the hospital	Home-ePRO at home
		(*N* = 113)	(*N* = 45)
Age	mean (SD, range)	45.1 years	58.7 years
		(SD 14.4, 22 to 81)	(SD 10.4, 29 to 74)
Sex	male	83.2 %	48.9 %
Diagnosis	gastrointestinal tumours	0.9 %	31.2 %
	glioma	15.7 %	-
	gynaecological tumours	0.9 %	33.3 %
	lung cancer	0.9 %	22.2 %
	neuroendocrine tumours	12.8 %	-
	testicular cancer	67.0 %	-
	other	1.8 %	13.3 %
Treatment	chemotherapy	5.3 %	100 %
	follow-up	88.5 %	-
	other	6.2 %	-
Device	iPad2	-	71.9 %
	own laptop or PC		28.1 %
Institution		Medical University of Innsbruck	Kufstein County Hospital

#### Home-ePRO patients

Of the 166 patients approached for participation at Kufstein County Hospital, 60 declined routine ePRO assessments at home; 55 chose autonomous data entry via the internet (home-ePRO; inclusion rate of 33.1 %); and 51 chose weekly phone calls. Owing to the particular nature of the phone interviews, these were excluded from evaluation. Regarding age, the two samples of refusing patients and those who chose phone calls were comparable but significantly older than patients participating in home-ePRO (*F* = 13.642, *p* < 0.001, 68.7/67.6 years [SD 10.5/9.2] versus 58.7 years [SD 10.7]). The groups did not differ in terms of patients’ sex, though a tendency that more men rejected ePRO and more women chose phone calls (*χ*2 = 4.778, *p* = 0.092) became apparent.

**Fig. 3:**
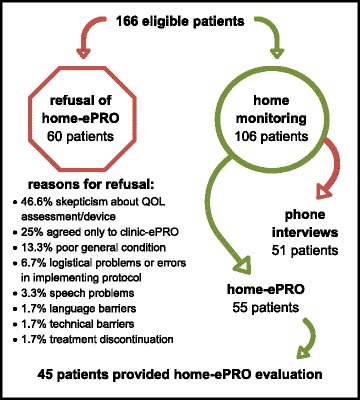
Patient flow for home-ePRO evaluation

The most frequent reason for refusal of QOL monitoring was skepticism about QOL assessment itself or reservations about the electronic device (46.6 %). Other reasons were exclusive agreement to clinic-ePRO at the hospital (25 %), poor general condition (13.3 %), logistical problems or errors in implementing protocol (6.7 %), speech problems (3.3 %), and in some rare cases, language barriers, technical barriers, or treatment discontinuation (1.7 % each) (see Fig. 3).

Of 55 patients participating in home-ePRO, four (7.2 %) died before evaluation, two (3.6 %) refused to evaluate the home-ePRO system, and four (7.2 %) did not respond to repeated mailings. In total, between March and August 2013, 45 patients (inclusion rate: 81.8 %, 27.1 % of all eligible patients) completed the home-ePRO evaluation form. The sex ratio was nearly balanced (51.1 % female); mean age was 58.7 years (SD 10.4, range 29 to 74), and pathology types were gynecological (33.3 %), gastrointestinal (31.2 %), lung (22.2 %), and lymphoma (13.3 %). The most frequent assessment mode was the iOS app (71.9 %). Detailed information on patient characteristics is shown in Table 1.

### Patients’ basic habits regarding their personal use of the internet

#### Clinic-ePRO patients

Four fifth (81.4 %) of the 113 clinic-ePRO patients stated to use the internet at home, most of them several times per week (66.4 %). Laptops (53.1 %), PCs (50.4 %) and smart phones (45.1 %) were the most often used internet-ready devices. Tablet PCs were used by only one fourth of patients (26.5 %) (multiple answers allowed for each patient). Nearly one fifth (18.6 %) stated not to pursue any internet activity (i.e. no internet use at all) and 11.5 % chose at least one activity. The majority of patients were quite familiar with the internet, as 28.3 % chose two to three activities and 41.6 % reported to use the internet for four or more activities. Reading and writing e-mails (82.2 %) was the by far most common activity on the internet, next to searching the internet for information (74.3 %), online banking or booking (72.6 %), entertainment (67.1 %), online shopping (45.1 %), and social media (40.7 %) (multiple answers allowed for each patient) (refer to Table 2). Approximately 8 % used the additional open answer to note that they use the internet for business matters.

**Table 2:** Patients’ basic habits regarding their personal use of the internet

		Agreement (%)	
		Clinic-ePRO	Home-ePRO
		*N* = 113	*N* = 45
Personal internet use	yes	81.4	77.8
Frequency of internet use^a^	several times per week	66.4	51.1
	several times per month	7.1	15.6
	less than once per month	8.8	6.7
	no internet use	15.0	20.0
	missing	2.7	2,2
Used internet-ready device^c^	laptop	53.1	51.1
	personal computer	50.4	35.6
	smart phone	45.1	8.9
	tablet-PC	26.5	20.0
Internet activities^c^	information search	74.3	71.1
	reading and writing e-mails^b^	82.2	53.3
	online shopping	45.1	31.1
	online banking/booking^b^	72.6	31.1
	entertainment^b^	67.1	28.9
	social media	40.7	15.6
Number of pursued internet activities	no internet activities	18.6	17.8
	1 activity	11.5	17.8
	2-3 activities	28.3	28.9
	4 or more activities	41.6	33.3

^a^ *N* = 152, as data from 6 patients is missing

^b^ *N* = 117, as data from 41 patients is missing

^c^Multiple answers allowed for each patient

#### Home-ePRO patients

Nearly four fifth (77.8 %) of the 45 home-ePRO patients patients stated to use the internet at home, about the half of them (51.1 %) several times per week. The most often used internet-ready device was laptops (51.1 %), followed by PCs (35.6 %) and tablet PCs (20.0 %). Smart phones were the least commonly used device for internet-access (8.9 %). Respectively almost 18 % of patients stated not to pursue any internet activity (i.e. no internet use at all) or at least one activity. The majority of home-ePRO patients were quite familiar with the internet, as 28.9 % chose two to three activities and 33.3 % reported to use the internet for four or more activities. Searching the internet for information (71.1 %) was the by far most common activity on the internet, next to reading and writing e-mails (53.3 %), online shopping and online banking or booking (31.1 %, respectively), entertainment (28.9 %) and social media (15.6 %) (multiple answers allowed for each patient) (refer to Table 2). Just a few home-ePRO patients (4.4 %) used the additional open answer to note that they use the internet for business matters.

### Patients’ attitudes towards clinic-ePRO/home-ePRO

#### Clinic-ePRO patients

Approximately two thirds of clinic-ePRO patients (64.6 %) indicated that they viewed PROs as a useful and adequate method to provide information on their QOL to their attending physicians. Nearly as many patients (61.1 %) were interested in seeing their own results. The vast majority of patients (94.7 %) would further participate in routine PRO assessment at the hospital and 68.1 % would opt for home-ePRO assessments. Of the latter, 43.4 % considered an annual interval to be sufficient. Most clinic-ePRO patients said they preferred electronic data entry for future completion of PRO measures; 23.9 % did not express a preference and seemed to be open-minded about clinic-ePRO (refer to Table 3).

**Table 3:** Patients’ attitude towards clinic-ePRO/home-ePRO

		Agreement (%)	
		Clinic-ePRO	Home-ePRO
		*N* = 113	*N* = 45
Considering PROs as an useful and adequate method to provide information on QOL to physicians		64.6	91.1
Wish to see own PRO results		61.1	77.8
Wish to discuss PRO results with treating physician^a^		-	82.2
Willingness to complete QLQ-C30	at the hospital	94.7	84.4
	at home^b^	68.1	-
For patients reasonable time frames for PRO completion at home	at least weekly	14.2	53.3
	monthly	18.6	24.4
	yearly	43.4	13.3
	missing	23.8	8.9
Preference of phone interview over home-ePRO^c^	no	97.3	93.2
Preferred mode of PRO assessment	paper-pencil	7.1	17.8
	electronic	67.2	60.0
	no preference	23.9	20.0
	decline of assessment	1.8	2.2

^a^ *N* = 45 patients at the Kufstein County Hospital

^b^ *N* = 113 patients at Medical University of Innsbruck

^c^ *N* = 118 patients (73 patients of Medical University of Innsbruck and 45 patients of the Department of Internal Medicine, Kufstein County Hospital)

#### Home-ePRO patients

The vast majority of home-ePRO patients (91.1 %) indicated that they viewed PROs as a useful and adequate method to provide information on their QOL to their attending physicians. There was apparently great interest in incorporating PRO data into the clinician-patient communication: 82.2 % of home-ePRO patients would appreciate a discussion of their results with their attending physician. Slightly more than half (53.3 %) of home-ePRO patients considered at least a weekly assessment interval for home monitoring reasonable; one fourth (24.4 %) stated a monthly interval to be sufficient. Electronic PRO assessment was preferred by 60 % over paper-pencil assessments. Those without a preference (20 %) might be open-minded towards an electronic assessment mode. Only a minority of home-ePRO patients (6.8 %) would prefer a regular phone call to autonomous data entry (refer to Table 3).

### Evaluation of the clinic-ePRO procedure

One hundred thirteen patients completed an evaluation form regarding the clinic-ePRO assessments conducted at Medical University of Innsbruck. Of these, 82.3 % reported no problems in questionnaire completion and 92.8 % experienced no difficulties in device handling. Only one patient had many problems and needed further assistance, which was provided by an accompanying relative. The large majority (91 %) of patients indicated that they were very satisfied with the graphic display of the questionnaire on the tablet screen. Only one suggestion for an improved design of the user interface was made, which was to include adjustable font sizes. Other ideas referred to construction of the response scale. Most patients (86.6 %) were highly satisfied with the level of privacy during PRO completion, and most (58.9 %) agreed that the always-available nature of clinic-ePRO was an advantage. The most frequently perceived disadvantages of clinic-ePRO were being too impersonal (57.5 %) and not allowing for individual situations (30.1 %). Technical issues and complexity were only rarely cited as a drawback (4.1 % combined) (refer to Table 4). One patient added a comment regarding data security concerns. Two patients, who agreed with the previously mentioned disadvantage of clinic-ePRO’s being too impersonal, made notes that paraphrased this answer category.

**Table 4:** Patients’ rating of feasibility of clinic-ePRO/home-ePRO

		Agreement (%)	
		Clinic-ePRO	Home-ePRO
		*N* = 113	*N* = 45
Did you have any difficulties completing the questionnaire?	not at all	82.3	97.8
	a little	14.2	2.2
	quite a bit	3.5	0.0
Did you have any difficulties handling the electronic device?	not at all	92.8	88.9
	a little	3.6	8.9
	quite a bit	2.7	2.2
	very much	0.9	0.0
Did you have any difficulties starting the app or entering your login data?^b^	not at all	-	72.7
	a little		27.3
How satisfied are you with the presentation of the questionnaire on the screen?	very much	91.0	95.5
	quite a bit	5.4	2.3
	not at all	3.6	2.3
How satisfied are you with the privacy during questionnaire completion?^a^	very much	86.6	-
	quite a bit	6.2	
	a little	2.7	
	not at all	4.5	
		Agreement (%)	
		Clinic-ePRO	Home-ePRO
		*N* = 113	*N* = 45
perceived advantages	no personal contact needed	11.0	6.7
	no drive to the hospital needed^b^	-	42.2
	availability of questionnaire at any time	58.9	44.4
	low cost	31.5	24.4
	feeling of being well cared for at home^b^	-	57.8
perceived disadvantages	handling of the internet is so difficult	0.0	1.4
	the technical requirements are too extensive	1.4	2.2
	does not come up to my individual situation	30.1	8.9
	too complex/confusing	2.7	0.0
	too impersonal	57.5	13.3
Overall, how satisfied are you with electronic quality of life assessment at home?^b^		Ø (SD, range)	9.1 (1.14, 5–10)

^a^ *N* = 113 patients at the Medical University of Innsbruck

^b^ *N* = 45 patients at the Kufstein County Hospital

### Evaluation of the home-ePRO procedure

At Kufstein County Hospital, 45 patients provided information on their experience with home-ePRO. Nearly three-quarters (72.7 %) reported no problems in accessing the website or iOS app or logging in. Six patients (13.3 %) indicated problems with remembering their login data, entering the URL of the website correctly, choosing correct buttons for navigation during the assessment, or issues regarding the SIM card of the provided iPad2. Such problems were solved easily during outpatient visits. Handling of the technical devices (the patient’s own PC or the provided iPad2) occurred without incident in 88.9 % of cases. Only one patient needed technical support for the iPad2 and received this assistance during an outpatient visit. Consistent with the very few reported problems, there were very few suggestions for necessary improvements. On a visual analog scale (ranging from 1 = “not at all satisfied” to 10 = “absolutely satisfied”), patients’ mean satisfaction with home-ePRO was 9.1 (SD 1.14, range 5 to 10).

Regarding advantages of home-ePRO, 57.8 % of patients appreciated most that they felt well cared for at home. Furthermore, patients perceived the always-available feature and independence from hospital visits as positive aspects of home-ePRO (44.4 % and 42.2 %, respectively). About a fourth of patients (24.4 %) reported the low cost of the system (24.4 %) to be beneficial. Only a few patients (6.7 %) rated the capability of providing PRO data without further hospital contact as advantageous.

Not a single patient experienced home-ePRO as too difficult or confusing. A small minority (13.3 %) felt that home-ePRO was too impersonal; 8.9 % thought that the assessment did not speak to their individual situation; and 2.2 % considered the technical requirements too extensive (refer to Table 4). There were no comments concerning other disadvantages.

## Discussion

This study reports on an evaluation of routine PRO assessments using a specialized software within two contexts: first, as an assessment of patients’ QOL before a follow-up/treatment appointment in a hospital setting (clinic-ePRO), and second, as regular monitoring of the symptom burden of chemotherapy outpatients in the home setting (home-ePRO).

The patients included in our study were relatively high experienced in internet use, in general open-minded towards ePRO assessments, rated them to be useful and would continue regular QOL-monitoring. CHES was well accepted and offered a user-friendly as well as feasible system for different assessment settings. In spite of this overall positive feedback, some previously identified implementation barriers of routine ePRO were replicated, and new ideas for further research areas could be generated.

As disclosed by both our study and the available literature, many important issues for successful implementation of ePRO in the routine clinical setting and at patients’ home can be identified. Efforts are needed not only to improve infrastructure for internet access, but also to encourage patients to use the internet for obtaining medical information and transmitting their health status and QOL information to providers [21]. Furthermore, to echo Rose and Bezjak [22], the person tasked with explaining the concept and implementation of QOL assessments, and instruments like home-ePRO in particular, is crucially important. Older people are particularly prone to have reservations concerning modern computer technology and need to be properly approached. This fact was demonstrated in our study when the most frequently cited reason for refusal of regular QOL-assessment was concern regarding the handling of an internet-enabled device. Simply providing user-friendly devices is not enough; older and/or computer-illiterate patients also need opportunities to familiarize themselves with the devices. Robben et al. [23] suggest another aspect that should be carefully considered, particularly among older patients: Aside from specific stand-alone training programs (such as heart-healthy eating or stress prevention strategies), web-based health applications should not be introduced without general information about the supplemental nature of these electronic tools. Fear of losing personal contact with health care professionals might deter patients from participating in home-ePRO, even if they are generally able to handle the necessary technology. Especially clinic-ePRO patients perceived the electronic assessments to be too impersonal and not coming up to their personal situation. Though we do not know how ePRO data was used by the clinicians of the Medical University of Innsbruck (it was available on their desktop PC), one might hypothesize that this negative perception of clinic-ePRO might relate to an insufficient integration of assessed data into the medical consultation. Further research needs to assess the use of ePRO data of health care professionals within the medical consultation and how this might influence patients’ perception of PROs.

As in other studies, most of our patients appreciated clinic-ePRO and were favorable to its extension from the hospital setting into their homes. Similarly to the results of Broering et al. [24], between 60 (home-ePRO) and 67 % (clinic-ePRO) of our patients stated a preference for an electronic mode of data collection for future PRO assessments. In addition, the vast majority of clinic-ePRO as well as home-ePRO patients (at least 97 per cent) did not perceive the assessment as too complex, difficult or the technical requirements as too extensive. Nonetheless, the phenomenon of a digital divide can be seen, especially for older patients, as patients who refused ePRO assessments at the Kufstein County Hospital or chose phone calls over home-ePRO were approximately 10 years older than home-ePRO participants. Those patients, who like to deal with new technologies and the internet, therefore, have a clear advantage over those who are not familiar with new media. Patients’ socioeconomic status may be of importance as well, and although technical progress continues to bring increasing affordability, future studies should also attend to this issue.

Some limitations of the study should be mentioned, as those restrict the generalizability of the reported results. First, some patients may have been especially motivated to participate in home-ePRO because of the provision of the iPad2, including the preinstalled iOS app. Because completion of the questionnaire was slightly more laborious via a web browser, one might hypothesize that without loaner devices, inclusion rates would have been lower. Second, an age bias emerged as older patients declined participation or preferred regular phone interviews to home-ePRO via an iPad2 or a website. It may be reasonable to assume that those patients also differed in terms of available internet equipment, user experience, and affinity for new media. Therefore, a digital divide may have put those patients at a disadvantage and their higher refusal rates may have whitewashed the overall study results, as only one third of all eligible patients agreed to use home-ePRO. Third, though the number of eligible patients and reasons for refusal were in detail assessed at Kufstein County Hospital, this was not the case at Medical University of Innsbruck. Consequently, we neither know how many patients were approached to participate in clinic-ePRO and its evaluation nor why they did not want to provide PRO-data or to evaluate the used ePRO software. Fourth, it should be noted that the clinic-ePRO group predominantly comprised a large number of relatively healthy and young testicular cancer patients. Those patients represent a quite specific sample of cancer patients, whose attitudes and opinions might differ from a mixed cancer patient sample undergoing active treatment. Overall, the results of our study primarily are based on data provided by patients, who seem to be especially open to ePRO from the outset. Further evaluation of CHES within a more heterogeneous sample of cancer patients should to be done.

## Conclusions

In line with other study results, our patients well accepted clinic-ePRO and home-ePRO and experienced it to be feasible. However, we must bear in mind that many patients declined to participate owing to computer illiteracy or worries about their own capability to handle the assessment device. To be more inclusive in the implementation of patient monitoring tools in daily clinical practice, the medical community will need to address several persistent doubts. Physicians as well as patients need further information about the complementary nature of PROs, which cannot replace the conventional medical consultation, but help to detect disturbing symptoms earlier, thereby allowing better structuring and more effective use of time in face-to-face encounters between patients and providers.

## References

[1] Pakhomov SV, Jacobsen SJ, Chute CG, Roger VL (2008). Agreement between patient-reported symptoms and their documentation in the medical record. Am J Manag Care.

[2] Fromme EK, Eilers KM, Mori M, Hsieh YC, Beer TM (2004). How accurate is clinician reporting of chemotherapy adverse effects? A comparison with patient-reported symptoms from the Quality-of-Life Questionnaire C30. J Clin Oncol.

[3] Weingart SN, Gandhi TK, Seger AC, Seger DL, Borus J, Burdick E (2005). Patient-reported medication symptoms in primary care. Arch Intern Med.

[4] Basch E, Jia X, Heller G, Barz A, Sit L, Fruscione M (2009). Adverse symptom event reporting by patients vs clinicians: relationships with clinical outcomes. J Natl Cancer Inst.

[5] Gravis G, Marino P, Joly F, Oudard S, Priou F, Esterni B (2014). Patients’ self-assessment versus investigators’ evaluation in a phase III trial in non-castrate metastatic prostate cancer (GETUG-AFU 15). Eur J Cancer.

[6] Hilarius DL, Kloeg PH, Gundy CM, Aaronson NK (2008). Use of health-related quality-of-life assessments in daily clinical oncology nursing practice: a community hospital-based intervention study. Cancer.

[7] Detmar SB, Muller MJ, Schornagel JH, Wever LD, Aaronson NK (2002). Health-related quality-of-life assessments and patient-physician communication: a randomized controlled trial. JAMA.

[8] Velikova G, Booth L, Smith AB, Brown PM, Lynch P, Brown JM (2004). Measuring quality of life in routine oncology practice improves communication and patient well-being: a randomized controlled trial. J Clin Oncol.

[9] Takeuchi EE, Keding A, Awad N, Hofmann U, Campbell LJ, Selby PJ (2011). Impact of patient-reported outcomes in oncology: a longitudinal analysis of patient-physician communication. J Clin Oncol.

[10] Atherton PJ, Sloan JA (2006). Rising importance of patient-reported outcomes. Lancet Oncol.

[11] Wintner LM, Giesinger JM, Kemmler G, Sztankay M, Oberguggenberger A, Gamper EM (2012). [The benefits of using patient-reported outcomes in cancer treatment: an overview]. Wien Klin Wochenschr.

[12] Gencer D, Tauchert F, Keilhauer N, Al-Batran SE, Stahl M, Oskay-Ozcelik G (2011). Cancer patients and the Internet: a survey of the ‘Quality of Life’ Working Groups of the Arbeitsgemeinschaft fur Internistische Onkologie and the Nord-Ostdeutsche Gesellschaft fur Gynakologische Onkologie. Onkologie.

[13] Jensen RE, Snyder CF, Abernethy AP, Basch E, Potosky AL, Roberts AC, Loeffler DR, Reeve BB: Review of Electronic Patient-Reported Outcomes Systems Used in Cancer Clinical Care. J Oncol Pract. 2014:e215-e222; published online on December 3, 2013. 10.1200/JOP.2013.001067 PMC4094646 24301843

[14] Bennett AV, Jensen RE, Basch E (2012). Electronic patient-reported outcome systems in oncology clinical practice. CA Cancer J Clin.

[15] McCleary NJ, Wigler D, Berry D, Sato K, Abrams T, Chan J (2013). Feasibility of computer-based self-administered cancer-specific geriatric assessment in older patients with gastrointestinal malignancy. Oncologist.

[16] Stukenborg GJ, Blackhall L, Harrison J, Barclay JS, Dillon P, Davis MA (2014). Cancer patient-reported outcomes assessment using wireless touch screen tablet computers. Qual Life Res.

[17] IKT-Einsatz in Haushalten 2014 [http://www.statistik.at/web_de/statistiken/informationsgesellschaft/ikt-einsatz_in_haushalten/]. Accessed at 25.03.2015.

[18] Holzner B, Giesinger JM, Pinggera J, Zugal S, Schopf F, Oberguggenberger AS (2012). The Computer-based Health Evaluation Software (CHES): a software for electronic patient-reported outcome monitoring. BMC Med Inform Decis Mak.

[19] Erharter A, Giesinger J, Kemmler G, Schauer-Maurer G, Stockhammer G, Muigg A (2010). Implementation of computer-based quality-of-life monitoring in brain tumor outpatients in routine clinical practice. J Pain Symptom Manage.

[20] Giesinger J, Kemmler G, Meraner V, Gamper EM, Oberguggenberger A, Sperner-Unterweger B (2009). Towards the implementation of quality of life monitoring in daily clinical routine: methodological issues and clinical implication. Breast Care (Basel).

[21] Wang JY, Bennett K, Probst J (2011). Subdividing the digital divide: differences in internet access and use among rural residents with medical limitations. J Med Internet Res.

[22] Rose M, Bezjak A (2009). Logistics of collecting patient-reported outcomes (PROs) in clinical practice: an overview and practical examples. Qual Life Res.

[23] Robben SH, Perry M, Huisjes M, van Nieuwenhuijzen L, Schers HJ, van Weel C (2012). Implementation of an innovative web-based conference table for community-dwelling frail older people, their informal caregivers and professionals: a process evaluation. BMC Health Serv Res.

[24] Broering JM, Paciorek A, Carroll PR, Wilson LS, Litwin MS, Miaskowski C: Measurement equivalence using a mixed-mode approach to administer health-related quality of life instruments. Qual Life Res. 2013;23:495–508. 10.1007/s11136-013-0493-7 23943258

